# Synthesis, Structure, and Photophysical Properties of Yellow-Green and Blue Photoluminescent Dinuclear and Octanuclear Copper(I) Iodide Complexes with a Disilanylene-Bridged Bispyridine Ligand

**DOI:** 10.3390/molecules26226852

**Published:** 2021-11-13

**Authors:** Toyotaka Nakae, Hiroto Miyabe, Masaki Nishio, Teppei Yamada, Yoshinori Yamanoi

**Affiliations:** Department of Chemistry, School of Science, The University of Tokyo, 7-3-1 Hongo, Bunkyo-ku, Tokyo 113-0033, Japan; nakae@chem.s.u-tokyo.ac.jp (T.N.); s2-6-hmiyabe@g.ecc.u-tokyo.ac.jp (H.M.); m-nishio@chem.s.u-tokyo.ac.jp (M.N.); teppei@chem.s.u-tokyo.ac.jp (T.Y.)

**Keywords:** disilanylene-bridged chelating ligand, copper(I) iodide, photoluminescence

## Abstract

The synthesis, structural, and photophysical investigations of CuI complexes with a disilanylene-bridged bispyridine ligand **1** are herein presented. Dinuclear (**2**) and ladder-like (**3**) octanuclear copper(I) complexes were straightforwardly prepared by exactly controlling the ratio of CuI/ligand **1**. Single-crystal X-ray analysis confirmed that dinuclear complex **2** had no apparent π…π stacking whereas octanuclear complex **3** had π…π stacking in the crystal packing. In the solid state, the complexes display yellow-green (*λ*_em_ = 519 nm, *Φ* = 0.60, *τ* = 11 µs, **2**) and blue (*λ*_em_ = 478 nm, *Φ* = 0.04, *τ* = 2.6 µs, **3**) phosphorescence, respectively. The density functional theory calculations validate the differences in their optical properties. The difference in the luminescence efficiency between **2** and **3** is attributed to the presence of π…π stacking and the different luminescence processes.

## 1. Introduction

Cu(I)-based emitters are considered as an attractive alternative to those containing platinum group metals for the development of luminescent materials because copper is abundant and inexpensive compared to other noble metals [1,2,3,4,5,6,7,8,9,10,11]. Due to their flexible coordination properties, Cu(I) halides-aggregates have been incorporated in coordination oligomers or polymers, which exhibit a range of photophysical properties [12,13,14,15]. Pyridine and its derivatives are one of the most investigated ligands for the copper(I) iodide complexes [16,17]. In general, the structural motifs of the core are dependent on the electronic and steric properties and stoichiometry of pyridine ligands, and the core structure affects the luminescent properties of the copper(I) iodide complexes. Besides the molecular structure, the intermolecular interaction is also critical for the luminescence properties of the copper(I) iodide complexes because strong intermolecular interactions, such as π…π stacking, in the solid state strongly suppress the luminescence as seen in copper iodide complexes with 2,2′-bipyridyl or 1,10-phenanthroline ligands [18,19,20,21,22,23]. Therefore, the control of the intermolecular interaction in copper(I) iodide complex is also crucial for efficient luminescent materials. 

We recently investigated aromatic compounds connected with disilanylene groups (‒SiR_2_SiR_2_‒) [24,25,26,27,28,29,30,31,32,33]. Disilanylene-linkers extend the conjugated system through σ–π interaction, similarly to C=C double bonds. On the other hand, the disilanylene moiety acts as a bulky group due to the tetrahedral geometry at silicon atoms and substituents on Si, and the single bond character of the Si‒Si bond allows the rotation along with the Si‒Si bond, which are different from the planar and rigid C=C double bond. These steric features of the disilanylene linker suppress the strong intermolecular π…π stacking and give unique properties, typically in the solid or aggregated states [34,35]. The use of disilanylene is a possible candidate for a linker moiety to control intermolecular interactions; the bulkiness will suppress strong intermolecular interaction and the long Si‒Si distance will give a large bite angle compared with the C‒C bond analogues. There are some complexes with disilanylene-bridged ligands, and they show unique structure and properties, such as σ-coordination of the Si‒Si bond [36], multiple switchable crystal polymorphs in a metal-organic framework [37], and photo-induced crystalline transformation [38]. However, the use of chelating disilane-based ligands was still limited.

A variety of inorganic building motifs have been reported for copper(I) with organic ligands. We herein report di- and octanuclear copper(I) iodide complexes with a disilaylene-bridged bispyridine bidentate ligand, 1,1,2,2-tetramethyl-1,2-di(pyridin-2-yl)disilane (**1**). The linker moiety in **1** is 1,1,2,2-tetramethyldisilanylene, which can act as a bulky linker as mentioned above. The copper(I) iodide complexes of **2** and **3** were selectively obtained by the stoichiometric control of copper(I) iodide and showed light blue and yellow-green photoluminescence in the solid state. To rationalize the different optical properties observed, quantum chemical calculations were performed for two copper clusters.

## 2. Results and Discussion

### 2.1. Synthesis and Crystal Structure of Cu Complexes **2** and **3**

**Figure 1:**
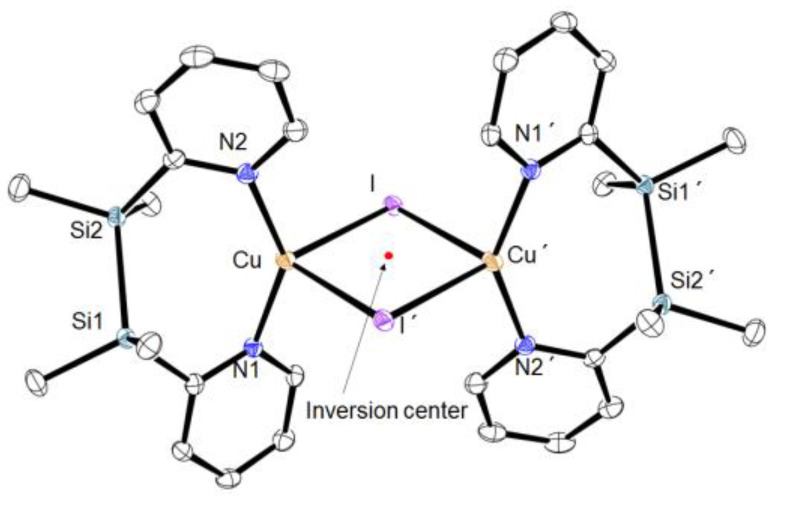
ORTEP drawing of complex **2** with thermal ellipsoids at the 50% probability level. Hydrogen atoms were omitted for clarity. Selected bond lengths and angles. Cu…Cu′: 3.8761(3) Å. Cu‒I: 2.7405(3) Å. Cu‒I′: 2.9689(3) Å. I…I′: 4.1982(3) Å. Cu‒N1: 1.987(2) Å. Cu‒N2: 1.993(2) Å. Si1‒Si2: 2.3441(7) Å. Cu…Si1: 3.3453(5) Å. Cu…Si2: 3.2611(5) Å. Cu‒I‒Cu′: 85.417(7)°. I‒Cu‒I′: 94.583(7)°. N1‒Cu‒N2: 140.45(6)°.

**Scheme 1:**
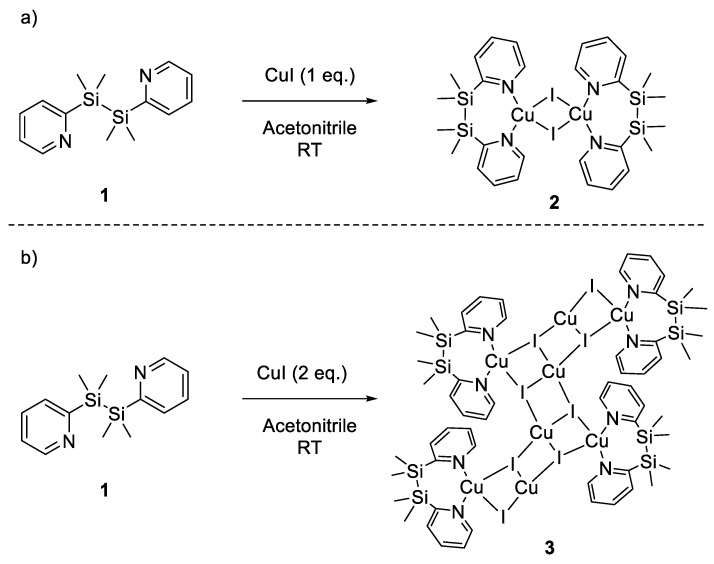
Reactions of bispyridine ligand **1** with copper iodide in 1:1 ratio (**a**) and 1:2 ratio (**b**).

The disilanylene-bridged bispyridine ligand **1** was synthesized by a reaction of 2-pyridyllithium with 1,2-dichloro-1,1,2,2-tetramethyldisilane. The structure was determined by ^1^H and ^13^C NMR (Figures S7–S10) and high-resolution mass spectroscopy. The dinuclear copper(I) iodide complex [Cu(µ-I)(**1**)]_2_ (**2**) was obtained by a reaction of copper(I) iodide and **1** in acetonitrile in a 1:1 ratio (Scheme 1a). The complex **2** was isolated as an air-stable pale-yellow solid. Single crystals were obtained by recrystallization from dichloromethane and *n*-hexane. Elemental analysis of **2** gave the expected composition. Single-crystal X-ray diffraction analysis revealed that the molecular structure consists of bimetallic iodo-bridged neutral complex [Cu(µ-I)(**1**)]_2_, and selected bond distances and angles are shown in Figure 1. The complex was crystallized in the triclinic space group *P*-1, and the asymmetric unit (half of the complex) was related with an inversion center. Two copper atoms were bridged by two iodo ligands, and each copper center was supported by a bidentate ligand **1** in an N_2_I_2_ distorted trigonal pyramidal geometry. The Cu1 atom positioned 0.315 Å out of the basal plane defined by N1, N2, and I1 toward I2. The torsion angle of the disilane moiety (C(pyridine)‒Si‒Si‒C(pyridine)) is 98.51(8)°, which indicates the staggered conformation of the disilane moiety. The distance of two copper atoms (3.8761(3) Å) was longer than the double of the covalent radius of copper (1.32 Å), which suggested the negligible direct Cu…Cu interaction [39,40]. The Si1‒Si2 distance (2.3441(7) Å) was in the normal range of Si‒Si bonds, and the Cu…Si distances were larger than the sum of the van der Waals radii of Cu and Si, suggestive of the absence of σ-bonding interaction between the Cu and Si‒Si bond [36]. The Cu‒I distances (2.7405(3) and 2.9689(3) Å) are significantly larger than those of related complexes with a Cu_2_I_2_ core [41,42,43,44]. The Cu‒N distances (1.987(2) and 1.993(2) Å) are slightly shorter than usual (2.02 Å for unsubstituted pyridine analogue), and the N1‒Cu‒N2 bite angle in the complex was 140.45(6)°, ideally 120° in a trigonal pyramidal geometry. This large bite angle possibly affects the long Cu‒I bond in the Cu_2_I_2_ core because it makes the distance between the methyl groups on the linker and iodide ligands shorten to induce the steric repulsion (the shorter C(Me)…I distance was 4.196(2) Å). The crystal packing pattern of **2** shows columns along with the *c*-axis. From the viewpoint of crystal packing, CH…π interactions were observed between methyl groups and pyridine rings, similarly to other disilyene-connected aromatic compounds (Figure S1) [45]. It is noteworthy that no significant π…π stacking was observed in the crystal packing (the intermolecular distance of adjacent pyridine-ring centroids: 4.31 Å), suggesting that the bulky linker moiety suppress the undesirable intermolecular π…π stacking as designed (Figure S3). We measured the powder XRD of **2**. The comparison of the diffraction pattern between the powder and single crystal of **2** is shown in Figure S6a. We did not observe any major changes in the diffraction pattern.

**Figure 2:**
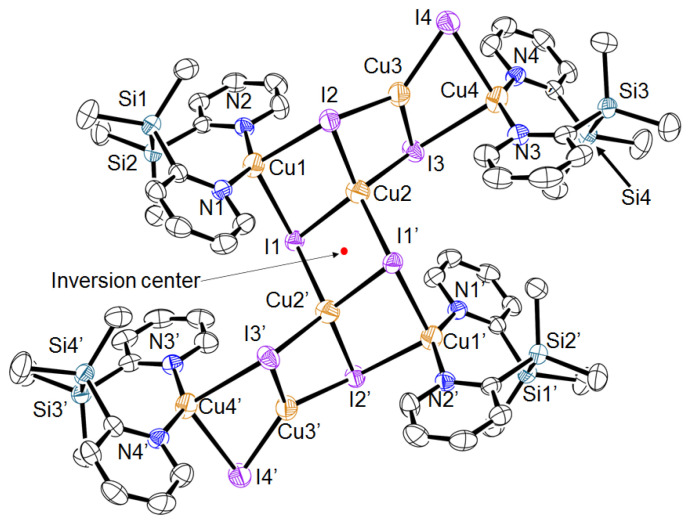
ORTEP drawing of complex 3 with thermal ellipsoids at the 50% probability level. Hydrogen atoms were omitted for clarity. Selected bond distances and angles. Cu1…Cu2, 3.0943(7) Å; Cu2…Cu3, 3.0365(6) Å; Cu3…Cu4, 2.9292(7) Å; Cu2…Cu2′, 3.5689(7) Å; Cu1‒I1, 2.8580(5) Å; Cu1‒I2, 2.9034(5) Å; Cu2‒I1, 2.6586(5) Å; Cu2‒I2, 2.6801(5) Å; Cu2‒I3, 2.6894(7) Å; Cu2‒I1′, 2.6801(6) Å; Cu3‒I2, 2.5644(7) Å; Cu3‒I3, 2.5440(5) Å; Cu3‒I4, 2.4996(5) Å; Cu4‒I3, 3.0754(5) Å; Cu4‒I4, 2.8532(5) Å; I1…I2, 4.5907(4) Å; I2…I3, 4.2253(5) Å; I3…I4, 4.4563(4) Å; I1…I1′, 3.9705(5) Å; Cu1‒N1, 1.964(3) Å; Cu‒N2, 1.966(3) Å; Cu4‒N3, 1.971(3) Å; Cu4‒N4, 1.965(3) Å; Si1‒Si2, 2.3498(14) Å; Si3‒Si4, 2.3424(14) Å; Cu1…Si1, 3.226(1) Å; Cu1…Si2, 3.247(1) Å; Cu4…Si3, 3.208(1) Å; Cu4…Si4, 3.253(1) Å; Cu‒I‒Cu′: 85.417(7)°. I‒Cu‒I′: 94.583(7)°. N1‒Cu1‒N2, 143.19(11)°; N3‒Cu4‒N4, 143.83(11)°.

Octacopper(I) iodide complex [Cu_4_I_4_(**1**)_2_] (**3**) was obtained by a reaction of ligand **1** and CuI in a 1:2 ratio (Scheme 1b). Elemental analysis of **3** gave the expected composition. The single crystals suitable for X-ray diffraction were obtained by recrystallization from tetrahydrofuran and *n*-hexane. Complex **3** crystallized in the monoclinic space group *P*2_1_/*c*. The crystal structure of **3** is shown in Figure 2, which can be interpreted by a dimer of a ladder [Cu_4_I_4_(**1**)_2_] cluster. In the asymmetric unit of complex **3**, three copper centers (Cu1, Cu2, and Cu4 in Figure 2) have a quasi-tetrahedral environment, and the other (Cu3) has quasi-trigonal planar geometry. These copper centers are bridged by three µ_3_-I and one µ_2_-I ligands, and the copper centers at the apical positions are supported by chelating ligand **1**. The Cu‒I bond distances of the trigonal planar copper(I) center (2.50–2.56 Å) are in the normal range of those of Cu‒I complexes [46,47,48]. Similarly to complex **2**, the Cu‒I bond lengths of copper centers supported by ligand **1** (Cu1‒I1, 2.8580(5) Å; Cu1‒I2, 2.9034(5) Å; Cu4‒I3, 3.0754(5) Å; Cu4‒I4, 2.8532(5) Å) are significantly longer than other reported complexes. Cu…Cu distances are also larger than the double of Cu(I) radius, suggestive of negligible Cu…Cu interaction. The bite angles (N1‒Cu1‒N2, 143.19(11)°; N3‒Cu4‒N4, 143.83(11)°) of ligand **1** are slightly larger than that of complex **2**. We also measured the powder XRD of **3**. The comparison of the diffraction pattern between the powder and single crystal is shown in Figure S6b. The PXRD resulted in the peaks closed to XRD being simulated by the single crystal, although anisotropic peaks are observed due to the interplanar π−π stacking in the crystalline packing of **3**.

Ladder-based copper iodide complexes with monodentate ligand generally gave coordination polymers, and some bulky pyridine ligands yielded tetracopper(I) iodide step complexes [49]. In the complexes with *N,N,N*-tridentate ligands, the ligands act as bridging ones for copper centers by η^2^-coordination for the apical position and η^1^-coordination for the side position [50,51,52,53]. In the case of the bidentate ligand **1**, the side-positioned copper atoms (Cu3 and Cu3′) are not supported by the chelating ligand. Presumably, the vacant site is capped by the iodide of another tetracopper(I) cluster to form complex **3**. To the best of our knowledge, this dimerization of the ladder copper(I) iodide core seems to be a new core architecture in octanucler copper(I) iodide complexes. The crystal packing pattern of **3** shows columns along with the *a*-axis similarly to complex **2** and intermolecular π…π stacking of pyridine rings along with the *b*-axis (the intermolecular distances of adjacent pyridine-ring centroids: 3.93 Å and 3.81 Å, Figures S2 and S4). All pyridine moieties in complex **3** are used for the stacking.

### 2.2. Photophysical Properties

**Figure 3:**
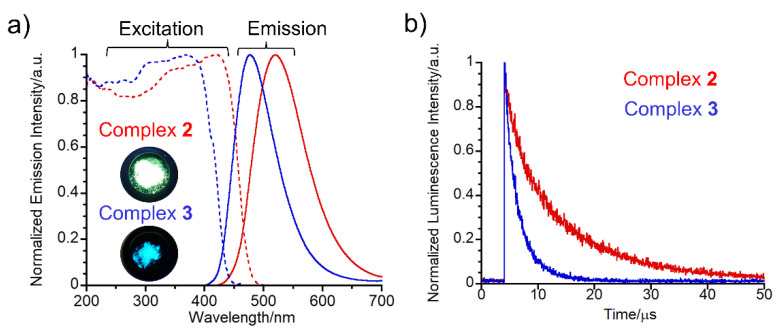
(**a**) Emission (solid lines, excited at 380 nm) and excitation (dashed lines, observed at 519 nm for **2** and 478 nm for **3**) spectra of **2** (red lines) and **3** (blue lines) in the solid state at room temperature. Photographs of **2** (top) and **3** (bottom) under 365 nm light irradiation. (**b**) Transient luminescence decay curves of **2** and **3** at room temperature.

We investigated the photophysical properties of **2** and **3** (Table 1). Figure 3 shows the excitation spectra, emission spectra, transient luminescence decay curves, and photographs under UV irradiation of **2** and **3** in the solid state. These Cu complexes did not display detectable photoluminescence in the solution state. This is in line with related copper-halide complexes, and it was ascribed to their chemical instability, owing to the flexible nature of the complex scaffold and ligand dissociation. Although most Cu(I) complexes with chelating pyridine-based ligands showed weak emission from the MLLCT excited state with low quantum yield, dicopper complex **2** showed broad and non-structured yellow-green (519 nm) emission in the solid state in good photoluminescent intensity (*Φ* = 0.60, Figure 2) [54]. The luminescence wavelength and quantum yield of **2** are in a normal range of the dicopper(I) iodide complexes with a rhombic core, which rationalizes the initial molecular design [55,56]. Complex **3** also shows broad and nonstructured blue emission (478 nm, *Φ* = 0.04) in the solid state. The emission wavelength of **3** is longer than that of the ladder-type polymer [CuI(4-picoline)]_∞_ (437 nm) [57] due to the lower LUMO levels induced by the introduction of the Si‒Si moiety [58,59,60]. The shapes of the spectra indicate the charge-transfer character of the emission. The lifetimes of the luminescence were 11 µs for **2** and 2.6 µs for **3**, respectively. The lifetimes in the order of µs suggest that the origins of the emissions are phosphorescence in both **2** and **3**.

### 2.3. Theoretical Consideration

**Figure 4:**
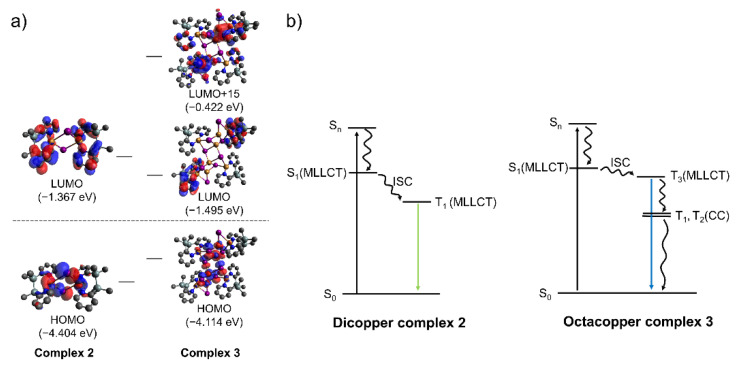
(**a**) Representative molecular orbitals and energy levels of **2** and **3** related to electronic transitions. (**b**) Plausible luminescent mechanisms of **2** and **3** at room temperature. Wavy lines indicate the nonradiative relaxation process.

Finally, we performed quantum chemical calculation based on density functional theory (DFT) to obtain some insight into the photophysical properties of complexes **2** and **3**. The structure optimizations were performed with a B3LYP functional and LANL2DZ (for iodide) and 6-31g(d) (for others) basis sets, where initial structures were obtained from the crystal structures. The highest-occupied molecular orbitals (HOMOs) of **2** and **3** are located on the copper iodide cores, and the lowest unoccupied MOs (LUMOs) are spread over ligand **1** (Figure 4a). It is noteworthy that complex **3** has higher HOMO energy and lower LUMO energy compared with complex **2**, although the emission wavelength of **3** is shorter than that of **2** at room temperature. 

To investigate the conflict of the energy gap and the emission wavelength in these complexes, we next performed time-dependent DFT (TD-DFT) calculations. Because complexes **2** and **3** showed phosphorescence, TD-DFT calculations were performed including triplet excitations (Figures S11 and S12 and Tables S2 and S3). The lowest energy singlet transitions of **2** and **3** were calculated as HOMO → LUMO excitations, which corresponded to metal-ligand-to-ligand charge-transfer (MLLCT) transitions. The lowest triplet excitations, which should be related to the emission for these complexes, were the corresponding MLLCT excited state for the dicopper(I) complex **2** but a cluster-centered (CC) excited state for the octacopper(I) complex **3** (for example, HOMO → LUMO + 15 in Figure 4a). The corresponding ^3^MLLCT excited states of **3** were found at higher energy than the CC excited states and the MLLCT excited state of **2**. In some copper iodide complexes, dual emission from similar ^3^MLLCT and ^3^CC excited states has been reported [61]. Therefore, both ^3^MMLCT and ^3^CC excited states in **3** are also potentially emissive.

From these calculations, the plausible luminescence mechanisms of **2** and **3** at room temperature are shown in Figure 4b. In dicopper(I) complex **2**, photoexcitation gives the lowest-energy singlet excited state ^1^MLLCT, which is relaxed to the ^3^MLLCT state via intersystem crossing (ISC) owing to the heavy atom effect of the copper iodide core. Because the ^3^MLLCT excited state is the lowest-energy triplet state in complex **2**, emission occurs from the excited state. Similarly to **2**, photoexcitation of **3** leads to the formation of the ^1^MLLCT and ^3^MLLCT state, from which phosphorescence at a shorter wavelength than **2** can occur. However, the almost ^3^MLLCT excited state would be relaxed to the lowest-energy triplet excited state, the ^3^CC excited state. Because only one emission was observed in emission spectrum of **3**, the ^3^CC excited state may be quenched at room temperature via a nonradiative process, such as vibrational relaxation due to the loose Cu‒I core structure. The related ladder-type copper iodide cluster also shows phosphorescence derived from ^3^MLLCT at room temperature and a longer phosphorescence wavelength is observed at lower temperatures, suggesting a low-lying potentially emissive ^3^CC excited state [62,63].

The partial quenching mechanism via the ^3^CC excited state would decrease the luminescent quantum yield of **3**. Therefore, the much lower quantum yield of **3** (*Φ* = 0.04) than that of **2** (*Φ* = 0.60) at room temperature could originate from the nonradiative relaxation of the lowest excited state. Another possible explanation of the decrease in the luminescence intensity of **3** is the intermolecular π…π stacking in the crystal packing. In general, when π…π stacking is present in the crystal packing, the excitation energy is delocalized among molecules, resulting in emission quenching through lattice defect [64,65,66]. The emission behavior of copper iodide clusters and their consideration in this study suggest the importance of the core architecture for emissive complexes. These mechanistic insights will provide a design for efficient luminescent materials based on copper iodide complexes.

## 3. Conclusions

In conclusion, we synthesized a disilane-bridged bispyridine ligand (**1**) and its dinuclear and octanuclear copper(I) iodide complexes (**2** and **3**) under the control of the stoichiometric ratio of **1** and CuI. Complexes **2** and **3** showed intense yellow-green (*λ*_em_ = 519 nm, *Φ* = 0.60, *τ* = 11 µs) and weak light blue (*λ*_em_ = 478 nm, *Φ* = 0.04, *τ* = 2.6 µs) photoluminescence in the solid state, respectively. Single-crystal X-ray diffraction analysis of **2** and **3** revealed that the bulkiness of the linker moiety in ligand **1** suppressed the intermolecular π…π interaction in **2** but did not suppress it in **3**. DFT and TD-DFT calculations suggest that the yellow-green emission of **2** originates from the lowest excited ^3^MLLCT state while the blue emission of **3** is not derived from the lowest-excited ^3^CC state but from a higher-lying ^3^MLLCT excited state. Thus, the weaker emission of **3** compared with that of **2** is due to quenching by the intermolecular π…π stacking and the nonradiative decay of the lowest excited state (^3^CC). These copper clusters with disilane-bridged bispyridine ligands in this work appear to be a promising tunable building block for application in photofunctional materials.

## Figures and Tables

**Table 1:** Luminescent properties of **2** and **3** in the solid state at room temperature.

Complex	2	3
*λ*_em_/nm	519	478
$∆\nu_{1/2}$/eV ^[a]^	0.46	0.43
*Φ* ^[b]^	0.60	0.04
*τ*/µs	11	2.6
*k*_r_ /s^−1 [c]^	5.5 × 10^4^	1.5 × 10^4^
*k*_nr_ /s^−1 [d]^	3.6 × 10^4^	3.7 × 10^5^

[a] Full width at half-maximum of the emission. [b] Absolute quantum yield excited at 380 nm in the solid state at room temperature. [c] Radiative rate constant calculated by *k*_r_ = *Φ*/*τ*. [d] Nonradiative rate constant calculated by *k*_nr_ = (1 − *Φ* )/*τ*.

## Data Availability

Data of the compounds are available from the author.

## Supplementary Materials

The following are available online. The supplementary materials include Experimental Section, Crystal structures of complexes **2** and **3**, NMR spectra of **1**, Crystallographic data for **2** and **3**, Results of quantum chemical calculations of **2** and **3**.

## Sample Availability

Samples of the compounds are available from the authors.
