# Supplementary material for: Synthesis, Structure, and Photophysical Properties of Yellow-Green and Blue Photoluminescent Dinuclear and Octanuclear Copper(I) Iodide Complexes with a Disilanylene-Bridged Bispyridine Ligand
Source: Molecules. 2021 Nov 13;26(22):6852. doi: 10.3390/molecules26226852 (PMC8617906; doi:10.3390/molecules26226852)
Supplement: Supplementary file 1 [file molecules-26-06852-s001.zip › molecules-1381871-supplementary.pdf]

Supporting Information

Synthesis, Structure and Photophysical Properties of Yellow-green and Blue Photoluminescent Dinuclear and Octanuclear Copper(I) Iodide Complexes with A Disilanylene-bridged Bispyridine Ligand

Toyotaka Nakae, Hiroto Miyabe, Masaki Nishio, Teppei Yamada, and Yoshinori Yamanoi *

Department of Chemistry, School of Science, The University of Tokyo, 7-3-1 Hongo, Bunkyo-ku, Tokyo 113-0033, Japan

Contents

1. **Experimental Section**
2. **Crystal Structures of Complexes 2 and 3**
3. **PXRD of 2 and 3**
4. **NMR Spectra of 1.**
5. **Crystallographic Data for 2 and 3**
6. **Results of Quantum Chemical Calculations**

1. Experimental Section

(1) General Comments

All manipulations were carried under an inert atmosphere. Reagents were purchased from commercial chemical suppliers and used without further purification. Solvents were dried according to standard procedures. ^{1}H and $^{13}C\{^{1}H\}$ nuclear magnetic resonance (NMR) spectra were recorded on a JEOL ECS400 spectrometer. ^{1}H and ^{13}C NMR spectra were referenced using the residual solvent peak ($CDCl_3$: ^{1}H = δ 7.26 ppm; ^{13}C = δ 77.16 ppm). GC-MS spectra were recorded with SHIMADZU GC-MS-QP2010 spectrometer. High-resolution ESI-TOF (electrospray ionization-time-of flight) mass spectra were recorded on a Waters LCT Premier XE spectrometer.

(2) Single Crystal X-ray Diffraction

X-ray diffraction data collection was carried out on using a Rigaku Saturn 724 (VariMax Dual) diffractometer with multi-layer mirror monochromated MoKα radiation (λ = 0.71073 Å) equipped with a PIRATUS 200K detector. The crystal-detector distance was 36 mm. The cell parameters were determined (Denzo software) from reflections taken from one set of 10 frames (1.0° steps in phi angle), each at 20 s exposure. The data were processed using Olex2 software package [1a]. The structures were solved by direct methods using the program SHELXT-2018/2 [1b]. The refinement and all further calculations were carried out using SHELXL-2018/3 [1c]. The hydrogen atoms were included in calculated positions and treated as riding atoms using SHELXL default parameters. The non-H atoms were refined anisotropically, using weighted full-matrix least-squares on F^2. A semiempirical absorption correction was applied using the MULscanABS routine in PLATON. These data can be obtained free of charge from The Cambridge Crystallographic Data Center via www.ccdc.cam.ac.uk/data_request/cif. The structures were deposited in Cambridge Structural Database and the numbers are 2106268 (**2**) and 2106304 (**3**).

(3) Powder X-ray Diffraction Measurement (PXRD)

PXRD measurements were performed at rt using a Rigaku SmartLab 3 kW diffractometer (Cu Kα, λ = 1.5405 Å). Crystalline powder was installed in a 0.7 mm glass capillary for X-ray diffraction. The data were collected at rt with a step of 0.02° (2θ) and a counting time of 0.2 s/step.

(4) Synthesis of 1,1,2,2-tetramethyl-1,2-di(pyridin-2-yl)disilane (1)

2-Bromopyridine (1.90 mL, 25 mmol) was dissolved in dry tetrahydrofuran (100 mL) and cooled to −78 °C. To the solution, *n*-butyl lithium in *n*-hexane (0.91 M, 23.0 mL, 21 mmol) was added dropwise over 15 min. The resulting red-orange suspension was stirred at the temperature for 30 min. The reaction mixture was added 1,2-dichloro-1,1,2,2-tetramethyldisilane (1.85 mL, 9.9 mmol) was added over 3 min. After stirred at the temperature for 1 h, water (3 mL) was added to quench the reaction. The mixture was allowed to warm up to room temperature. To the resulting pale-yellow suspension was added brine (50 mL) and then extracted with diethyl ether (50 mL) three times. The yellow solution was dried over Na_2SO_4, filtered, and evaporated to give orange oil. The crude product was purified by flash column chromatography (neutralized Al_2O_3, *n*-hexane/ethyl acetate = 9/1). The middle colorless band (R_f = 0.58) was collected and evaporated to dryness. Colorless white powder was obtained (0.903 g, 33%). ^{1}H NMR (400 MHz, $CDCl_3$) 8.72 (2H, dd, 6-py), 7.50 (2H, td, 5-py), 7.38 (2H, d, 3-py), 7.12 (2H, td, 4-py), 0.40 (12H, s, Me). ^{13}C{^{1}H} NMR (100 MHz, $CDCl_3$) 168.0 (2-py), 150.1 (6-py), 133.8 (3-py), 129.6 (4-py), 122.2 (5-py), −4.3 (Me). HR-ESI-TOF-MS: calcd for $[M + Na]^+$, 295.1063; Found, 295.1043.

(5) Synthesis of 2

CuI (95.6 mg, 0.50 mmol) and **1** (137 mg, 0.50 mmol) were dissolved in CH_3CN (10 mL) at room temperature. The resulting yellow mixture was stirred at room temperature overnight. The solvent was evaporated under reduced pressure, and the residue was recrystallized from dichloromethane and *n*-hexane to give **2** as pale-yellow crystals (181 mg, 78%). Yellow block-shaped single crystals suitable for X-ray diffraction measurements were obtained by the recrystallization from *n*-hexane and CH_2Cl_2. IR (selected, cm^{-1}) 3050, 2953, 2890, 1582, 1557, 1455, 1413, 1271, 1247, 1163, 1137, 1092, 1051, 1014, 826, 796, 790, 778, 731, 714, 664, 649. Anal. Calcd for $C_{28}H_{40}Cu_2I_2N_4Si_4$: C, 36.32; H, 4.35; N, 6.05. Found: C, 36.11; H, 4.23; N, 5.94.

(6) Synthesis of 3

CuI (98.3 mg, 0.52 mmol) and **1** (69.9 mg, 0.26 mmol) were dissolved in CH_3CN (10 mL) at room temperature. The resulting yellow mixture was stirred at room temperature overnight. The solvent was evaporated under reduced pressure and the residue was reprecipitated from acetonitrile/diethyl ether to give **3** as pale-brown powder (102 mg, 61%). Colorless single crystals suitable for X-ray diffraction measurements were obtained by the recrystallization from *n*-hexane and tetrahydrofuran. IR (selected, cm^{-1}) 3077, 3053, 2951, 2892, 1583, 1559, 1421, 1408, 1270, 1405,

1270, 1246, 1160, 1138, 1091, 1053, 1017, 834, 793, 733, 724, 711, 643. Anal. Calcd for $C_{56}H_{80}Cu_8I_8N_8Si_8$: C, 25.74; H, 3.09; N, 4.29. Found: C, 25.49; H, 3.11; N, 4.23.

(7) Quantum Chemical Calculations

Quantum chemical calculations were performed using Gaussian 16 Rev. C01 program [2]. The initial structures of complexes **2** and **3** were obtained from the crystal structures and optimized by using B3LYP as a functional at LANL2DZ for iodide and 6–31 g(d,p) for other levels of theory. Frequency analyses were performed for the optimized structures to confirm local minima of the energy. Time-dependent DFT calculations were performed by using optimized structures. The calculated electronic transitions of **2** and **3** were presented in Figures S9–S10 and Tables S2–S3.

(8) References

[1] (a) Dolomanov, O. V.; Bourhis, L. J.; Gildea, R. J.; Howard, J. A. K.; Puschmann, H. *J. Appl. Cryst.* **2009**, *42*, 339–341; (b) Sheldrick, G. M. *Acta Cryst.* **2015**, *A71*, 3–8; (c) Sheldrick, G. M. *Acta Cryst.* **2015**, *C71*, 3–8.

[2] Gaussian 16, Revision C.01, Frisch, M. J.; Trucks, G. W.; Schlegel, H. B.; Scuseria, G. E.; Robb, M. A.; Cheeseman, J. R.; Scalmani, G.; Barone, V.; Petersson, G. A.; Nakatsuji, H.; Li, X.; Caricato, M.; Marenich, A. V.; Bloino, J.; Janesko, B. G.; Gomperts, R.; Mennucci, B.; Hratchian, H. P.; Ortiz, J. V.; Izmaylov, A. F.; Sonnenberg, J. L.; Williams-Young, D.; Ding, F; Lipparini, F.; Egidi, F.; Goings, J.; Peng, B.; Petrone, A.; Henderson, T.; Ranasinghe, D.; Zakrzewski, V. G.; Gao, J.; Rega, N.; Zheng, G.; Liang, W.; Hada, M.; Ehara, M.; Toyota, K.; Fukuda, R.; Hasegawa, J.; Ishida, M.; Nakajima, T.; Honda, Y.; Kitao, O.; Nakai, H.; Vreven, T.; Throssell, K.; Montgomery, Jr. J. A.; Peralta, J. E.; Ogliaro, F.; Bearpark, M. J.;Heyd, J. J.; Brothers, E. N.; Kudin, K. N.; Staroverov, V. N.; Keith, T. A.; Kobayashi, R.; Normand, J.; Raghavachari, K.; Rendell, A. P.; Burant, J. C.; Iyengar, S. S.;Tomasi, J.; Cossi, M.; Millam, J. M.; Klene, M.; Adamo, C.; Cammi, R.; Ochterski, J. W.; Martin, R. L.; Morokuma, K.; Farkas, O.; Foresman, J. B.; Fox, D. J. Gaussian, Inc., Wallingford CT, 2019.

2. Crystal Structures of 2 and 3

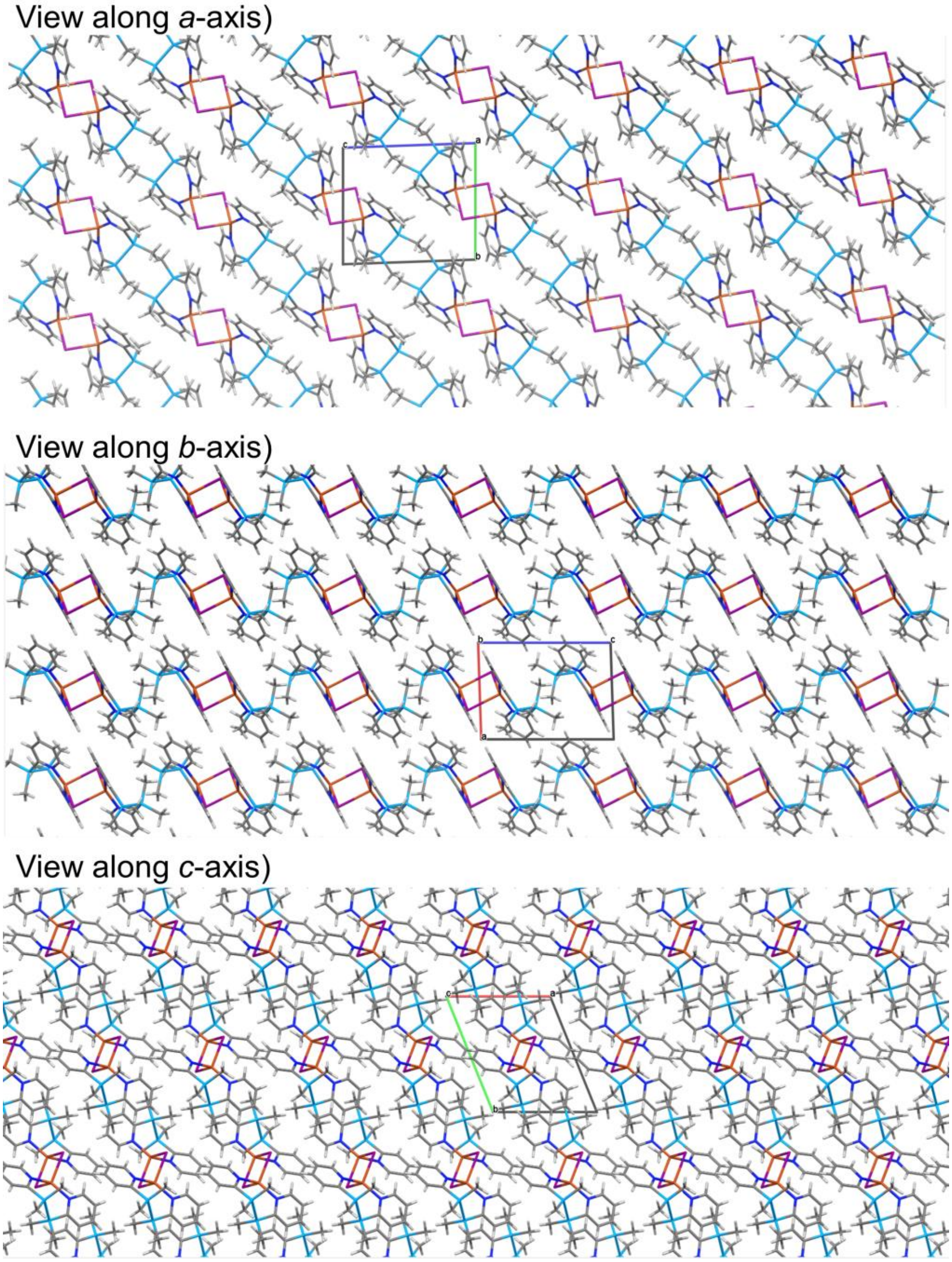

Figure S1. Crystal packing views of **2** along top) *a*-, middle) *b*- and bottom) *c*-axes.

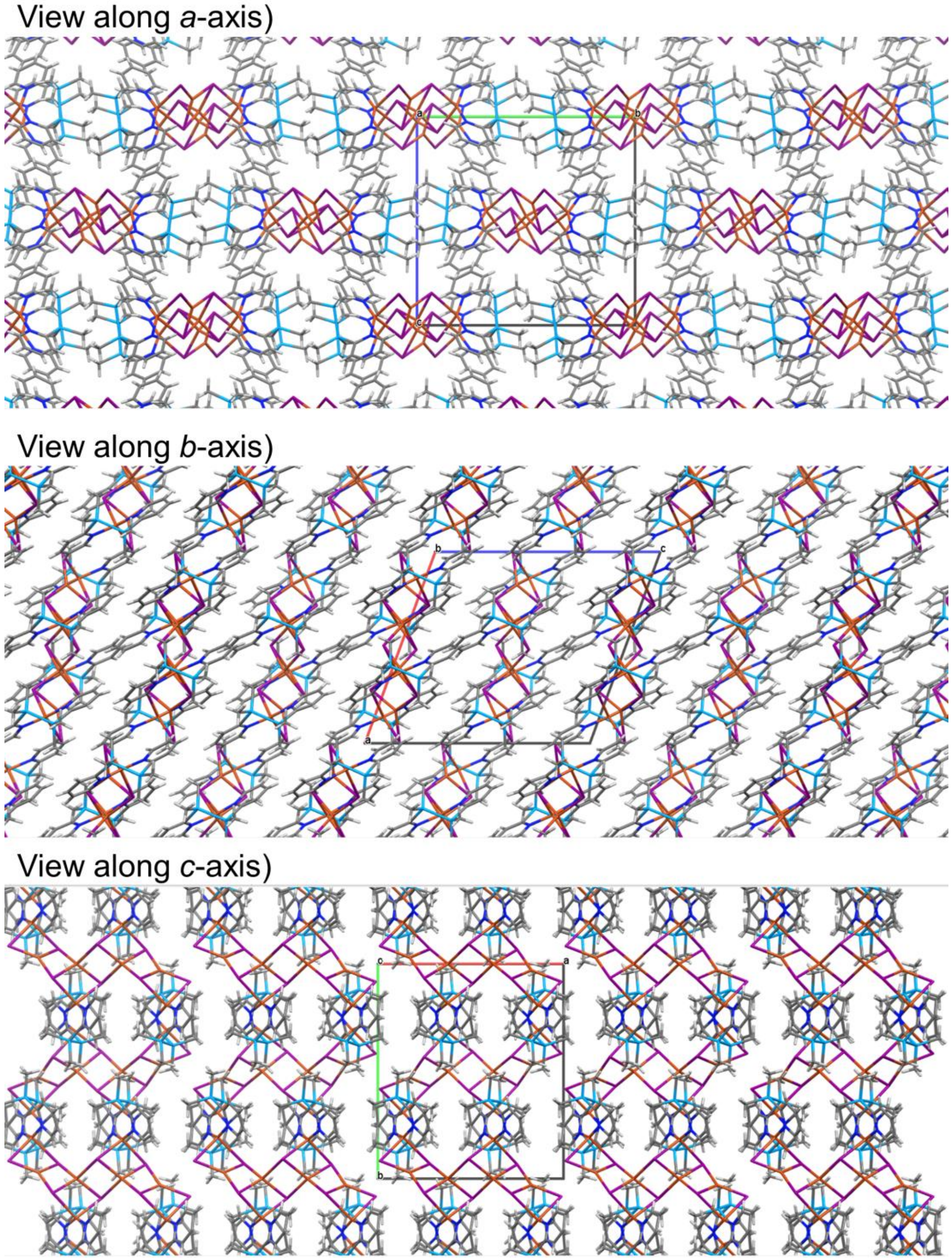

Figure S2. Crystal packing views of **3** along top) *a*-, middle) *b*- and bottom) *c*-axes.

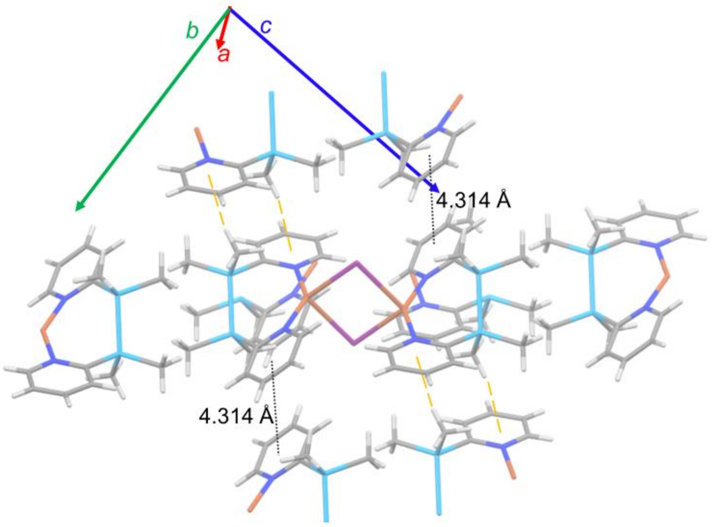

Figure S3. Packing environment of a molecule of complex **2** in crystal. Orange dashed lines represent intermolecular CH…π interactions. Values besides the black dotted lines indicate the shortest distance of the two centroids of the pyridine rings. The distances were calculated by Olex2 [1a].

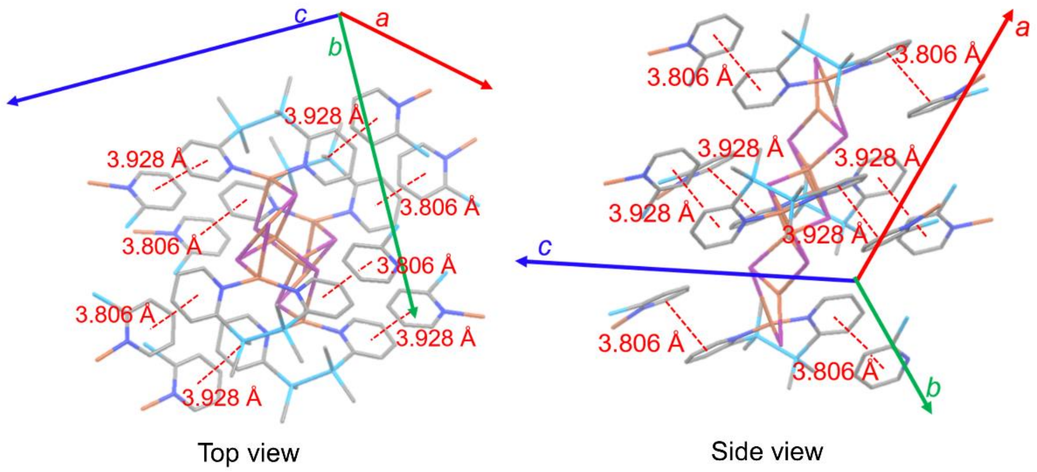

Figure S4. Packing environment of a molecule of complex **3** in crystal. Red dotted lines represent intermolecular π…π stacking of the pyridine rings. Values besides the dotted lines indicate the distances of the two centroids of the pyridine rings. The distances were calculated by Olex2 [1a].

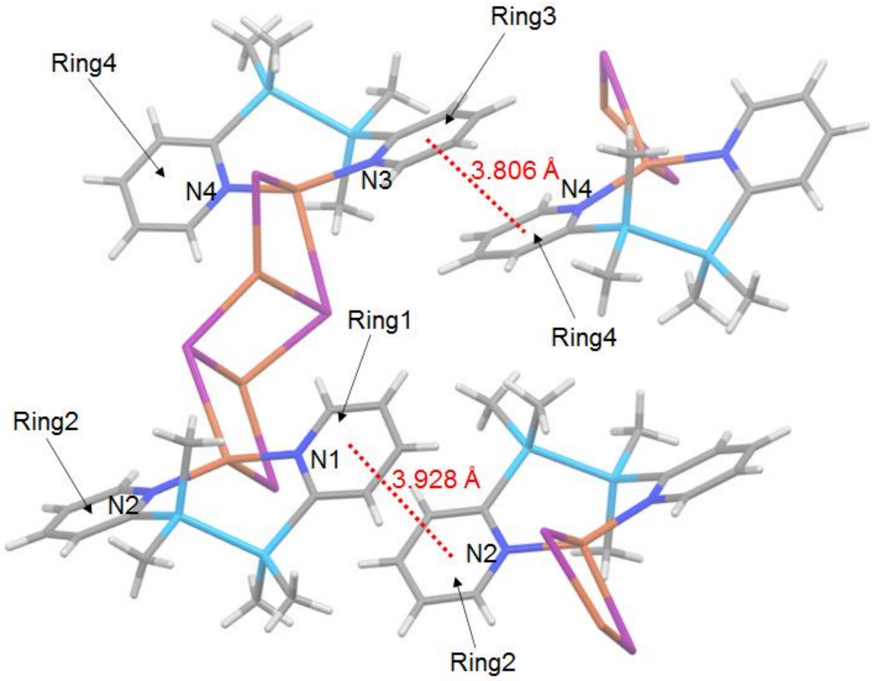

	Interplanar angle/°	Centroid-to-centroid distance/Å	Intercentroid shift/Å
Ring1…Ring2[a]	4.705	3.928	1.960
Ring3…Ring4[b]	2.746	3.806	1.700

Figure S5. The angles between two aromatic rings of π…π interactions of **3** calculated by Olex2 [1a]. [a] Ring1 represents the pyridine-ring containing N1 atom, and Ring2 is the pyridine-ring containing N2 atom in another molecule. [b] Ring3 represents the pyridine-ring containing N3 atom, and Ring4 is the pyridine-ring containing N4 atom in another molecule.

3. PXRD of 2 and 3

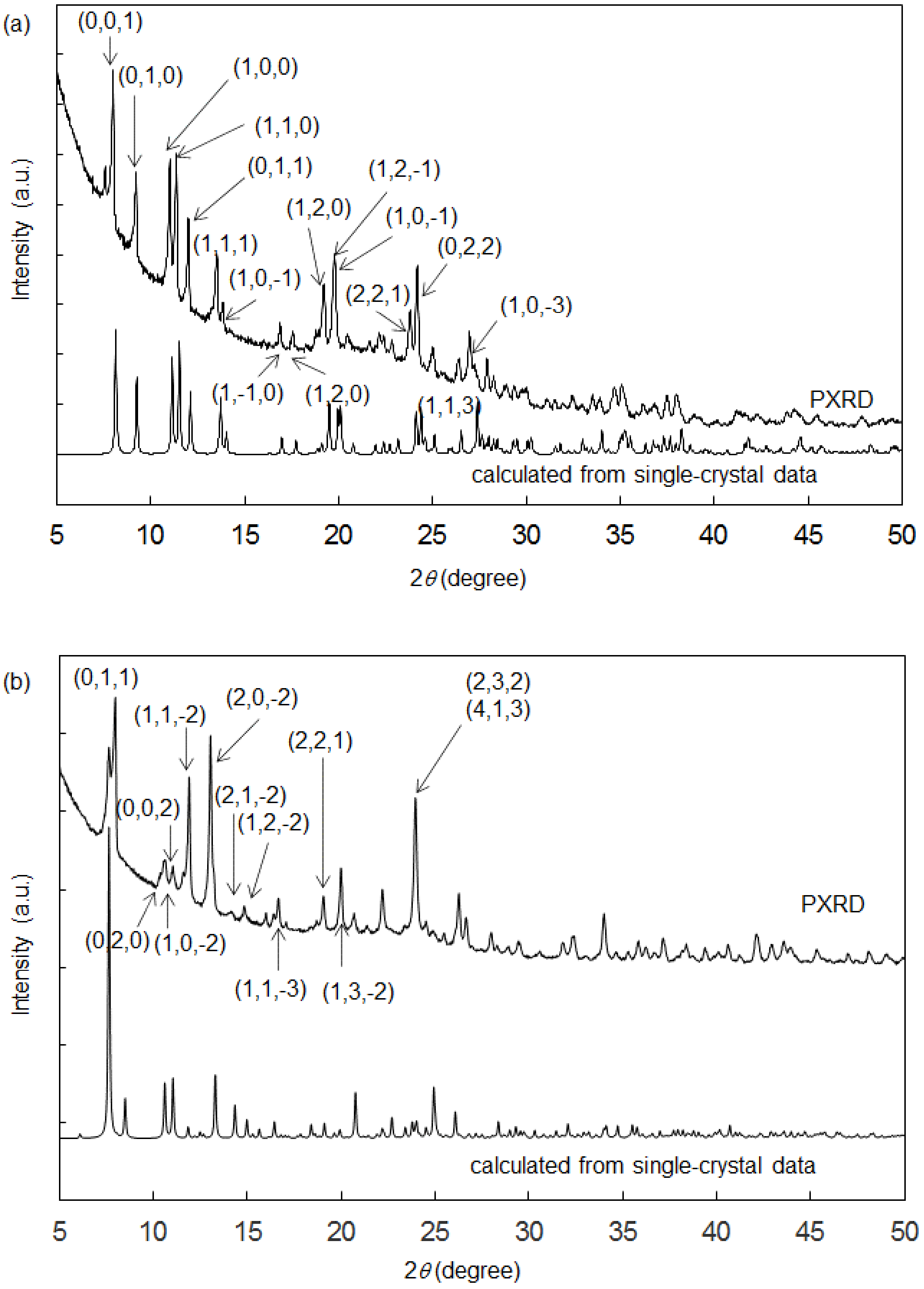

Figure S6. PXRD measured at rt and XRD pattern calculated by single crystal data. (a) Complex **2**. (b) Complex **3**.

4. NMR Spectra of 1

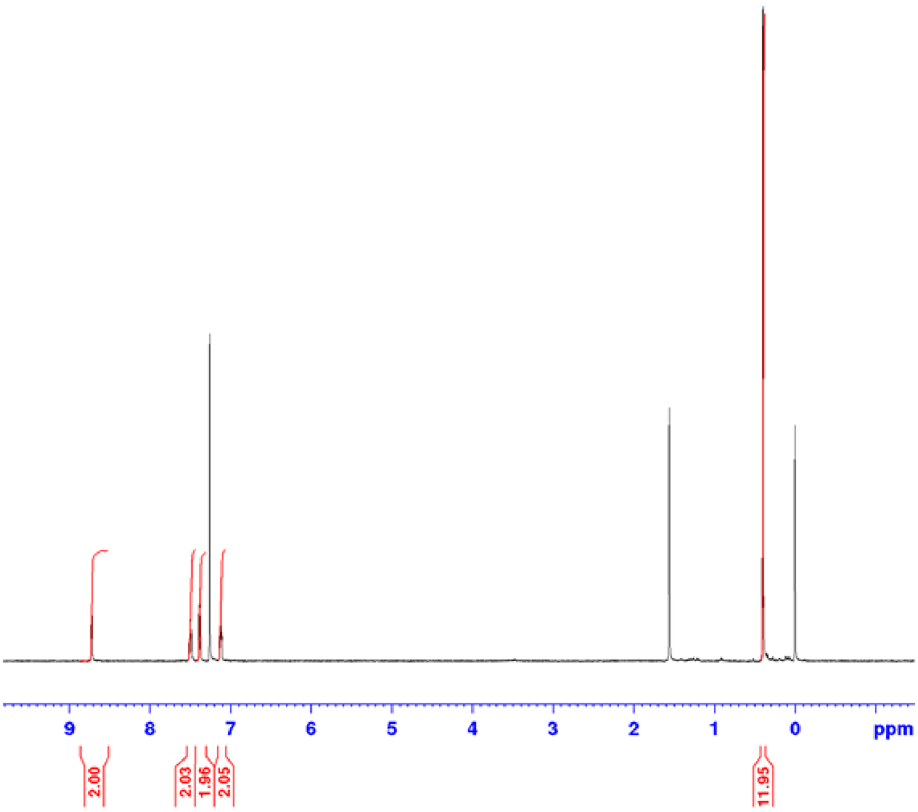

Figure S7. ^{1}H NMR (400 MHz, $CDCl_3$, rt) of **1**.

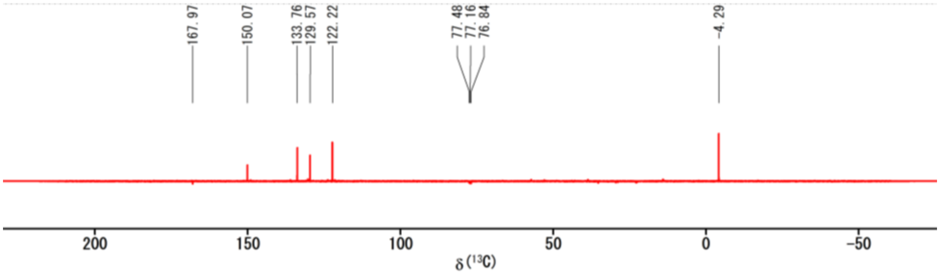

Figure S8. ^{13}C(APT) NMR (100 MHz, $CDCl_3$, rt) of **1**.

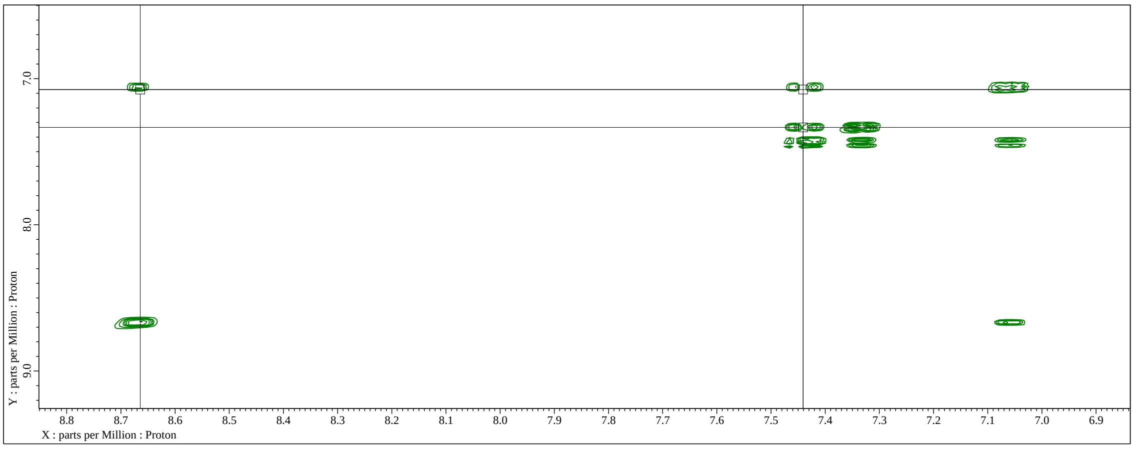

Figure S9. ^{1}H-^{1}H COSY spectrum of **1**.

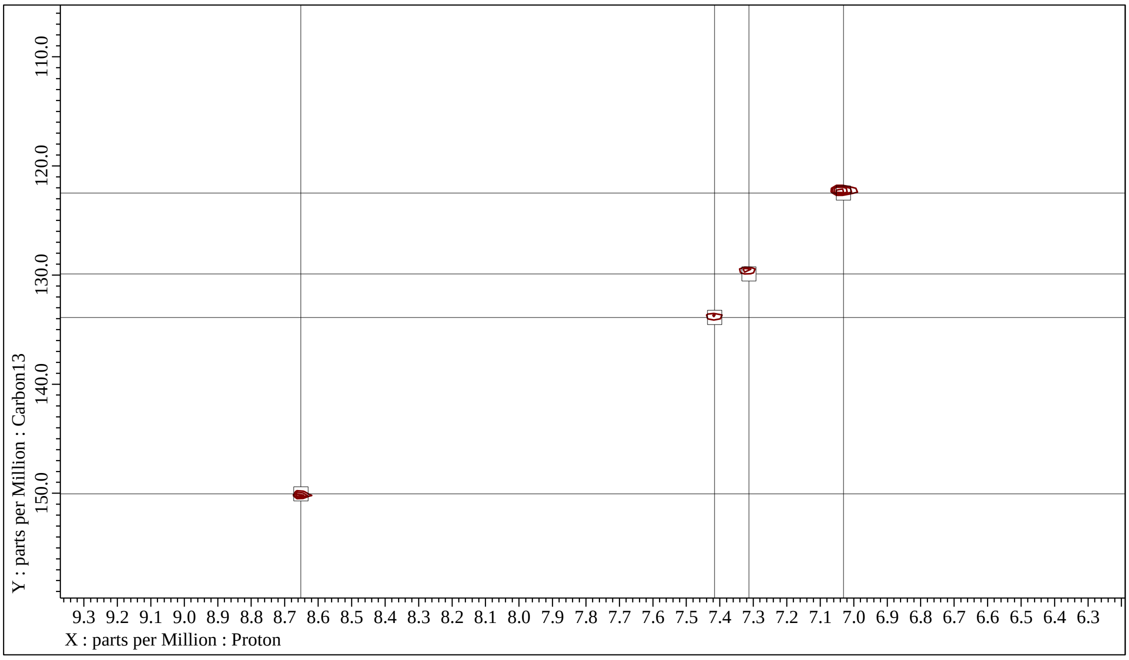

Figure S10. ^{1}H-^{13}C HSQC NMR spectrum of **1**.

5. Crystallographic Data for 2 and 3

Table S1. Crystallographic data for complexes **2** and **3**.

	**2**	**3**
Empirical formula	$C_{28}H_{40}Cu_2I_2N_4Si_4$	$C_{56}H_{80}Cu_8I_8N_8Si_8$
Formula weight	925.88	2613.52
Temperature/K	93	263
Wavelength/Å	0.71073	0.71073
Crystal system	Triclinic	Monoclinic
Space group	*P*-1(#2)	$P2_1/c$ (#14)
a/Å	8.5631(2)	15.4834(4)
b/Å	10.3035(2)	16.7861(3)
c/Å	10.8667(2)	17.0971(4)
α/°	87.089(2)	90
β/°	87.212(2)	110.230(3)
γ/°	67.951(2)	90
V/Å^3	887.05(3)	4169.52(18)
Z	1	2
D_{calcd}/Mg m^{-3}	1.733	2.082
μ(Mo Kα)/mm^{-1}	3.099	5.114
F(000)	456.0	2480
Crystal size/mm^3	0.278 × 0.278 × 0.089	0.15 × 0.13 × 0.05
Reflections collected	24553	26076
Independent reflections	4281	8602
Completeness to θ	100.00% (θ = 26.32°)	99.92% (θ = 25.30°)
Max. and min. transmission	1.000 and 0.450	1.000 and 0.610
No. of data/restraints/parameters	4281/0/185	8602/3/405
Goodness of fit on F^2	1.078	1.061
R_1 [$I > 2\sigma(I)$]	0.0177	0.0276
wR_2 (all data)	0.0415	0.0652
Largest diff. peak and hole (e Å^{-3})	0.79 and −0.44	1.08 and −0.81

6. Results of Quantum Chemical Calculations

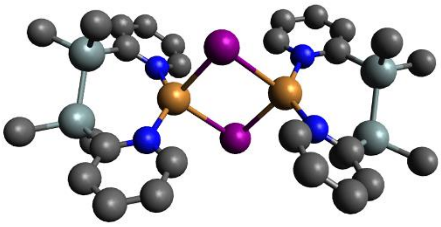

Figure S11. Optimized structure of complex **2** with the B3LYP functional and the LANL2DZ for iodine and 6-31G(d) for others basis sets. Hydrogen atoms are omitted for clarity.

Serial number	Atomic number	*x*/Å	*y*/Å	*z*/Å
1	53	−0.47743	−0.18712	2.198149
2	29	2.349559	−0.03225	0.154575
3	14	5.194347	0.877213	−0.86461
4	14	5.323744	−1.00311	0.56473
5	7	2.925661	1.721198	0.543051
6	7	2.690915	−1.89696	0.263455
7	6	4.104247	2.174474	0.018651
8	6	4.484786	3.50864	0.197343
9	1	5.421878	3.856727	−0.22599
10	6	4.584373	0.504949	−2.61473
11	1	4.639926	1.410108	−3.23228
12	1	3.550731	0.147531	−2.64488
13	1	5.225632	−0.25484	−3.07974
14	6	3.984514	−2.33322	0.274856
15	6	6.943231	1.617121	−1.02558
16	1	7.613604	0.890609	−1.49999
17	1	7.381355	1.892161	−0.05923
18	1	6.936049	2.511531	−1.66158
19	6	5.207586	−0.46816	2.381637
20	1	6.001654	0.24963	2.622633
21	1	5.327755	−1.33328	3.044944
22	1	4.247323	0.002686	2.612119
23	6	3.676895	4.384503	0.923376
24	1	3.973485	5.419841	1.068634
25	6	2.148646	2.566041	1.251679
26	1	1.239886	2.134366	1.6632

27	6	4.268405	−3.69981	0.179745
28	1	5.301567	−4.03235	0.176662
29	6	2.491312	3.896526	1.468168
30	1	1.835931	4.528089	2.059738
31	6	7.019975	−1.83264	0.315191
32	1	7.154923	−2.21604	−0.70299
33	1	7.163742	−2.66643	1.013752
34	1	7.821945	−1.10949	0.50457
35	6	3.231591	−4.63092	0.107054
36	1	3.446638	−5.69412	0.039864
37	6	1.691674	−2.79765	0.206598
38	1	0.68868	−2.38784	0.237196
39	6	1.91887	−4.16857	0.137317
40	1	1.074664	−4.84955	0.101995
41	53	0.477389	0.186311	−2.19819
42	29	−2.34956	0.032154	−0.1545
43	14	−5.19462	−0.87653	0.86463
44	14	−5.3235	1.003607	−0.56499
45	7	−2.92604	−1.72123	−0.54275
46	7	−2.69051	1.896912	−0.26358
47	6	−4.10475	−2.17417	−0.01835
48	6	−4.48555	−3.50829	−0.19684
49	1	−5.42274	−3.85611	0.226495
50	6	−4.58467	−0.50411	2.614726
51	1	−4.64042	−1.40916	3.232416
52	1	−3.55096	−0.14688	2.644873
53	1	−5.22581	0.255879	3.079581
54	6	−3.98401	2.333456	−0.27514
55	6	−6.94367	−1.61604	1.025633
56	1	−7.61392	−0.8893	1.49988
57	1	−7.3818	−1.89115	0.059302
58	1	−6.93672	−2.51034	1.661792
59	6	−5.20729	0.468403	−2.38182
60	1	−6.0015	−0.24923	−2.6228
61	1	−5.3272	1.333475	−3.04524
62	1	−4.24711	−0.00269	−2.61215

63	6	−3.67779	−4.38444	−0.92267
64	1	−3.97458	−5.41974	−1.06776
65	6	−2.14915	−2.56635	−1.25119
66	1	−1.24028	−2.13493	−1.66273
67	6	−4.26761	3.700124	−0.1802
68	1	−5.3007	4.032885	−0.17725
69	6	−2.49207	−3.8968	−1.46747
70	1	−1.83678	−4.52861	−2.05888
71	6	−7.01958	1.833542	−0.31573
72	1	−7.15454	2.217124	0.702382
73	1	−7.16309	2.667266	−1.01443
74	1	−7.82169	1.110548	−0.50507
75	6	−3.2306	4.631012	−0.1075
76	1	−3.44542	5.694269	−0.04044
77	6	−1.69108	2.797398	−0.20673
78	1	−0.68817	2.38736	−0.23721
79	6	−1.91798	4.168368	−0.1376
80	1	−1.07362	4.849164	−0.10227

LUMO + 4 (−0.687 eV)	HOMO (−4.404 eV)
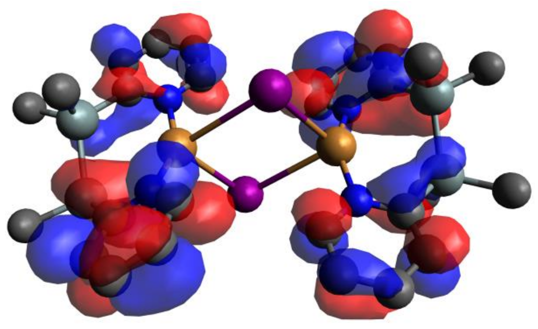	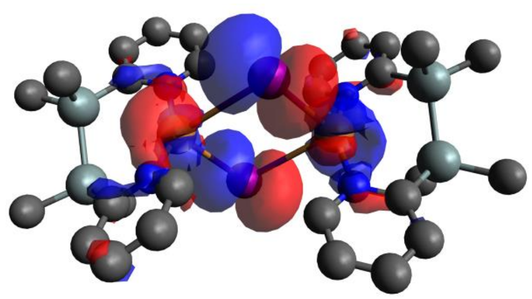
LUMO + 3 (−1.101 eV)	HOMO − 1(−4.603 eV)
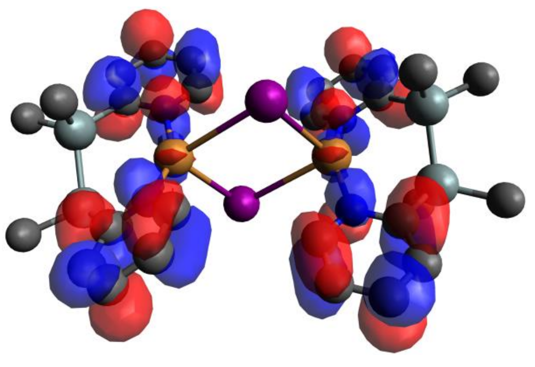	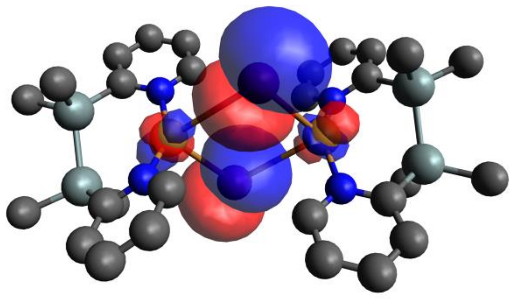
LUMO + 2 (−1.127 eV)	HOMO − 2 (−4.606 eV)

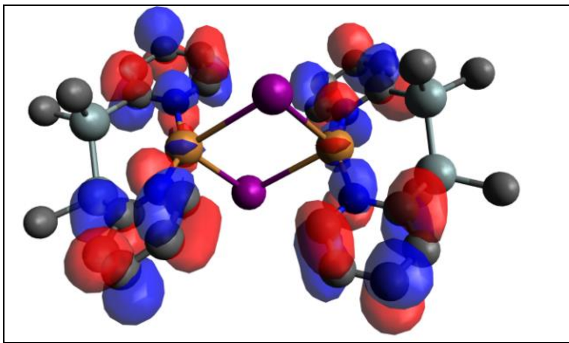	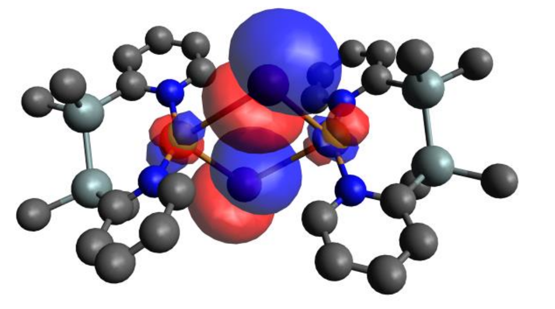
LUMO + 1 (−1.348 eV)<br>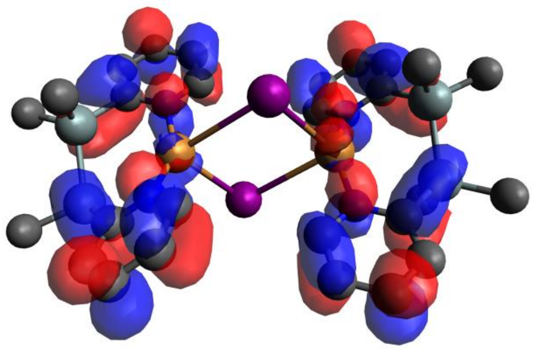	HOMO – 3 (−4.858 eV)<br>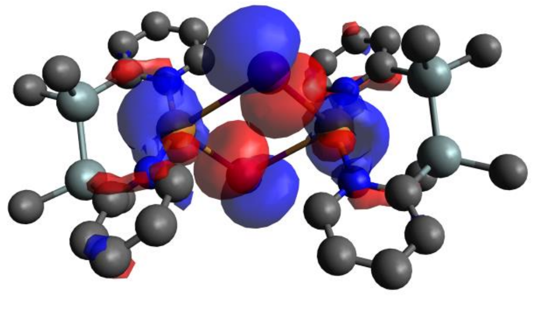
LUMO (−1.367 eV)<br>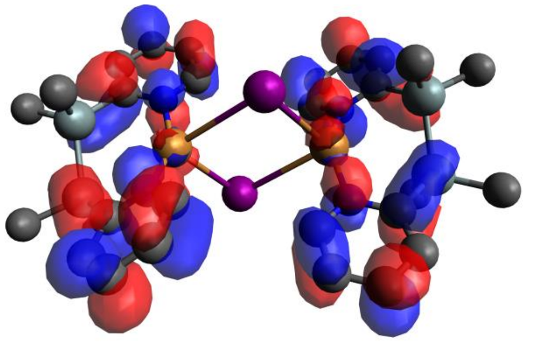	HOMO – 4 (−4.897 eV)<br>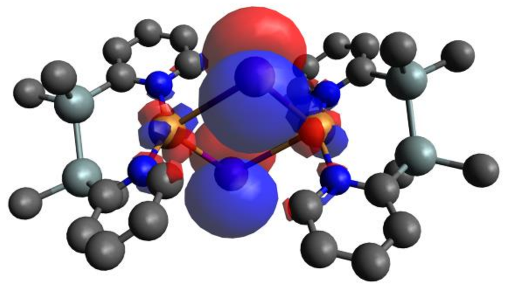

Table S2. Calculated electronic transitions of the dicopper complex **2** obtained by TD-DFT calculation at the B3LYP/LANL2DZ (for iodine) and 6-31G(d) (for others) level.

State[a]	Component (coefficient)	*E*/eV	*λ*/nm	*f*
T1	H − 2 → L + 1 (0.38507)<br>H − 2 → L + 2 (0.10503)<br>H → L (0.55187)<br>H → L + 3 (0.10821)	2.0754	597.39	0.0000
T2	H − 2 → L (0.40441)<br>H → L + 1 (0.53497)<br>H → L + 2 (0.12844)	2.0802	596.03	0.0000

T3	H − 2 → L + 4 (0.37395)<br>H → L + 1 (−0.16778)<br>H → L + 2 (0.54981)	2.3357	530.82	0.0000
T4	H − 2→ L + 3 (0.39945)<br>H→ L (−0.14062)<br>H→ L + 3 (0.53520)	2.3453	528.64	0.0000
S1	H − 2 → L + 1 (0.27847)<br>H → L (0.60831)<br>H → L + 3 (−0. 10307)	2.3653	524.18	0.0288
S2	H − 2 → L (0.30233)<br>H → L + 2 (0.58437)<br>H → L + 3 (−0.14467)	2.3676	523.67	0.0000
S3	H − 2 → L + 3 (0.31547)<br>H → L + 1 (0.12517)<br>H → L + 2 (0.57546)	2.5116	493.64	0.0000
S4	H − 4 → L (0.10294)<br>H − 2 → L + 2 (0.35225)<br>H → L (0.11207)<br>H → L + 3 (0.55748)	2.5247	491.09	0.0459
T5	H − 5 → L + 1 (−0.14217)<br>H − 4 → L (−0.10999)<br>H − 3 → L + 1 (0.13320)<br>H − 1 → L (0.61299)	2.5562	485.03	0.0000
S5	H − 5 → L + 1 (−0.10627)<br>H − 1 → L (0.67966)<br>H → L (0.10158)	2.6161	473.93	0.0098

[a] S and T represents singlet and triplet excited states, respectively.

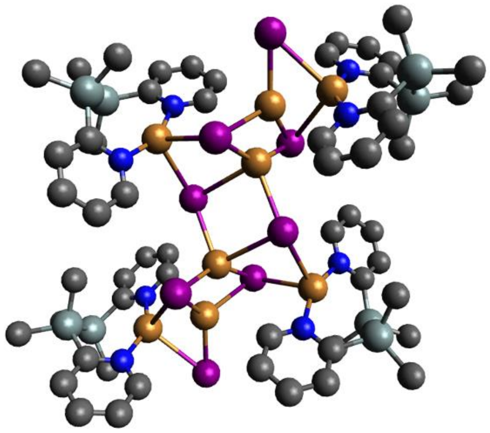

Figure S12. Optimized structure of complex **3** with the B3LYP functional and the LANL2DZ for iodine and 6-31G(d) for others basis sets. Hydrogen atoms are omitted for clarity.

Serial number	Atomic number	*x*/Å	*y*/Å	*z*/Å
1	53	−0.21281	−2.06956	0.809812
2	53	−2.3921	−0.77579	−2.91931
3	53	−3.41255	1.093858	1.684362
4	53	−6.66901	−0.00486	−2.01385
5	29	−2.04772	−3.47821	−1.24509
6	29	−5.34942	2.543723	−0.75515
7	29	−4.25436	0.451969	−1.0438
8	29	−2.10005	0.377688	−0.53077
9	14	−5.93398	5.482732	0.374767
10	14	−3.12942	−6.31579	−0.5845
11	14	−2.95865	−5.95022	−2.91225
12	14	−6.31376	5.349985	−1.95761
13	7	−0.85411	−4.15262	−2.55272
14	7	−3.5723	−3.5909	−0.13698
15	7	−4.31113	3.422569	−2.10145
16	7	−6.57725	2.772638	0.733411
17	6	−1.24528	−5.21348	−3.31922
18	6	−4.01362	−4.83339	0.22788
19	6	−6.79768	4.019286	1.246082
20	6	−0.40949	−5.69317	−4.33196
21	1	−0.73058	−6.53724	−4.93445
22	6	−4.74817	4.570142	−2.70327
23	6	1.206844	−4.00991	−3.77823

24	1	2.155109	−3.50004	−3.91078
25	6	−7.63957	4.191794	2.35086
26	1	−7.80497	5.189668	2.744199
27	6	0.827765	−5.09061	−4.56877
28	1	1.479558	−5.46001	−5.35634
29	6	0.337371	−3.56972	−2.78437
30	1	0.596371	−2.73143	−2.14674
31	6	−5.06269	−4.96722	1.140732
32	1	−5.40555	−5.95917	1.417948
33	6	−4.0207	5.131406	−3.75481
34	1	−4.37972	6.042007	−4.22458
35	6	−5.66719	−3.83556	1.691156
36	1	−6.4829	−3.93427	2.402352
37	6	−2.40168	3.375351	−3.5616
38	1	−1.48424	2.868734	−3.83801
39	6	−7.18648	1.717638	1.306211
40	1	−6.99673	0.753847	0.848048
41	6	−4.15965	−2.50326	0.391524
42	1	−3.79554	−1.5332	0.062377
43	6	−4.33725	−4.8324	−3.5731
44	1	−5.31161	−5.31762	−3.43361
45	1	−4.2053	−4.6515	−4.64664
46	1	−4.37269	−3.86048	−3.07294
47	6	−3.17305	2.851186	−2.53164
48	1	−2.88065	1.941117	−2.02111
49	6	−5.20667	−2.58269	1.303466
50	1	−5.63226	−1.66532	1.693558
51	6	−8.26652	3.094459	2.940641
52	1	−8.92238	3.224717	3.797357
53	6	−8.03608	1.832826	2.402096
54	1	−8.50325	0.943844	2.813018
55	6	−1.44648	−6.61238	0.239642
56	1	−0.9645	−7.4975	−0.1948
57	1	−1.57378	−6.79363	1.313889
58	1	−0.7657	−5.76416	0.121058
59	6	−2.8362	4.533505	−4.19414

60	1	−2.26461	4.971095	−5.00814
61	6	−4.08867	5.495106	0.804204
62	1	−3.57981	4.58073	0.486942
63	1	−3.94647	5.599374	1.886644
64	1	−3.59033	6.34325	0.318264
65	6	−7.85018	4.346443	−2.42725
66	1	−7.74499	3.282617	−2.19212
67	1	−8.03887	4.430938	−3.50461
68	1	−8.73639	4.722971	−1.90156
69	6	−3.097	−7.61929	−3.82006
70	1	−2.31105	−8.32641	−3.53028
71	1	−3.04716	−7.48347	−4.90787
72	1	−4.06305	−8.08734	−3.59679
73	6	−6.51487	7.100532	−2.67456
74	1	−7.39821	7.586191	−2.24315
75	1	−6.66292	7.065666	−3.76127
76	1	−5.65066	7.743249	−2.47139
77	6	−6.69857	7.087794	1.056238
78	1	−6.24564	7.956029	0.563009
79	1	−6.51265	7.194549	2.132413
80	1	−7.7809	7.143089	0.891719
81	6	−4.18823	−7.86705	−0.26712
82	1	−5.2026	−7.77374	−0.67179
83	1	−4.26963	−8.08202	0.805928
84	1	−3.72274	−8.74101	−0.73817
85	53	0.212814	2.069562	−0.80981
86	53	2.392097	0.775793	2.919306
87	53	3.412553	−1.09386	−1.68436
88	53	6.669006	0.004861	2.013845
89	29	2.047723	3.478208	1.245085
90	29	5.349417	−2.54372	0.755152
91	29	4.254364	−0.45197	1.043798
92	29	2.100053	−0.37769	0.530765
93	14	5.933982	−5.48273	−0.37477
94	14	3.12942	6.315788	0.584502
95	14	2.958645	5.950215	2.912254

96	14	6.31376	−5.34999	1.957605
97	7	0.85411	4.152615	2.552717
98	7	3.572298	3.590898	0.136976
99	7	4.311132	−3.42257	2.101445
100	7	6.577245	−2.77264	−0.73341
101	6	1.245276	5.213483	3.319216
102	6	4.013616	4.833393	−0.22788
103	6	6.797679	−4.01929	−1.24608
104	6	0.409491	5.693165	4.331955
105	1	0.730583	6.537236	4.934447
106	6	4.748172	−4.57014	2.703271
107	6	−1.20684	4.009914	3.778233
108	1	−2.15511	3.500039	3.910782
109	6	7.639571	−4.19179	−2.35086
110	1	7.804973	−5.18967	−2.7442
111	6	−0.82777	5.090613	4.568765
112	1	−1.47956	5.460014	5.356344
113	6	−0.33737	3.569715	2.784369
114	1	−0.59637	2.731432	2.146742
115	6	5.062685	4.967217	−1.14073
116	1	5.405546	5.959166	−1.41795
117	6	4.020696	−5.13141	3.754808
118	1	4.379724	−6.04201	4.22458
119	6	5.66719	3.835564	−1.69116
120	1	6.482899	3.934269	−2.40235
121	6	2.401675	−3.37535	3.561596
122	1	1.484239	−2.86873	3.83801
123	6	7.186484	−1.71764	−1.30621
124	1	6.996732	−0.75385	−0.84805
125	6	4.15965	2.503264	−0.39152
126	1	3.79554	1.533202	−0.06238
127	6	4.33725	4.832398	3.5731
128	1	5.311614	5.317618	3.433611
129	1	4.205296	4.651504	4.646644
130	1	4.372693	3.860476	3.072941
131	6	3.173049	−2.85119	2.531641

132	1	2.880652	−1.94112	2.021108
133	6	5.206668	2.582686	−1.30347
134	1	5.632261	1.665317	−1.69356
135	6	8.266524	−3.09446	−2.94064
136	1	8.922381	−3.22472	−3.79736
137	6	8.036079	−1.83283	−2.4021
138	1	8.503245	−0.94384	−2.81302
139	6	1.44648	6.612384	−0.23964
140	1	0.964502	7.4975	0.194798
141	1	1.573777	6.793625	−1.31389
142	1	0.765698	5.76416	−0.12106
143	6	2.836199	−4.53351	4.194138
144	1	2.264612	−4.9711	5.008144
145	6	4.088671	−5.49511	−0.8042
146	1	3.579813	−4.58073	−0.48694
147	1	3.946474	−5.59937	−1.88664
148	1	3.590332	−6.34325	−0.31826
149	6	7.850175	−4.34644	2.427253
150	1	7.744985	−3.28262	2.192115
151	1	8.038871	−4.43094	3.504613
152	1	8.736391	−4.72297	1.901561
153	6	3.097001	7.619292	3.820056
154	1	2.311049	8.326409	3.530277
155	1	3.047162	7.483469	4.90787
156	1	4.063053	8.087339	3.596793
157	6	6.514871	−7.10053	2.674559
158	1	7.398214	−7.58619	2.243145
159	1	6.662917	−7.06567	3.761269
160	1	5.650657	−7.74325	2.471392
161	6	6.698565	−7.08779	−1.05624
162	1	6.245643	−7.95603	−0.56301
163	1	6.512649	−7.19455	−2.13241
164	1	7.780895	−7.14309	−0.89172
165	6	4.188232	7.867052	0.267121
166	1	5.202603	7.773735	0.671788
167	1	4.269628	8.082017	−0.80593

168	1	3.722735	8.741011	0.738172

LUMO + 15 (−0.422 eV) 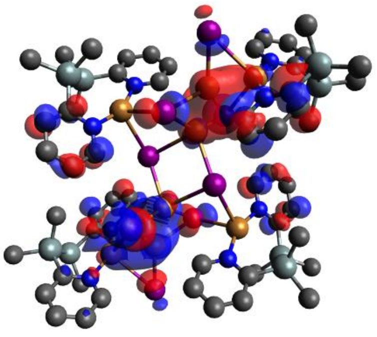	LUMO + 2 (−1.287 eV) 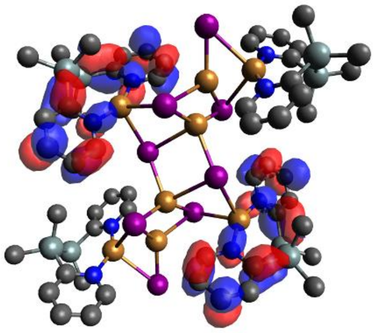
LUMO + 14 (−0.439 eV) 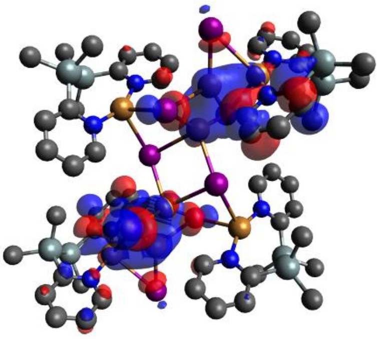	LUMO + 1 (−1.492 eV) 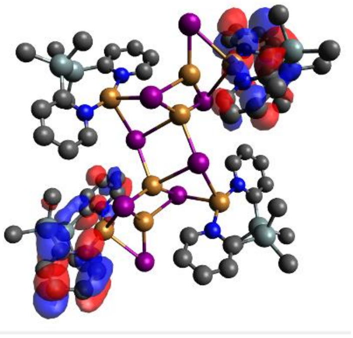
LUMO + 13 (−0.494 eV) 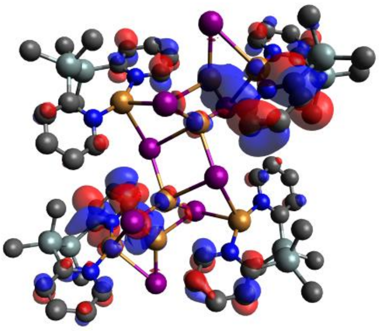	LUMO (−1.495 eV) 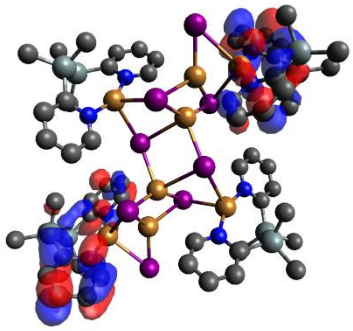
LUMO + 12 (−0.501 eV)	HOMO (−4.114 eV)

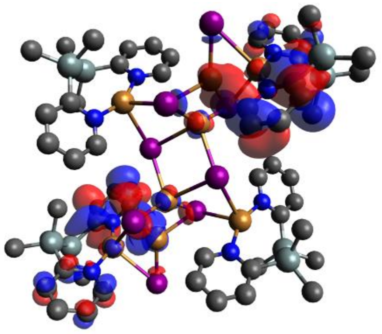	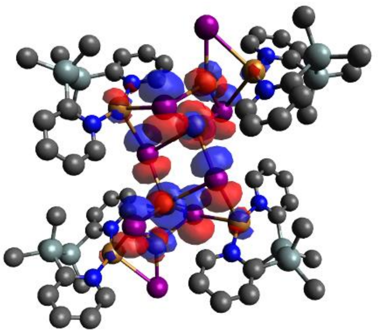
LUMO + 11 (−0.549 eV)	HOMO − 1 (−4.165 eV)
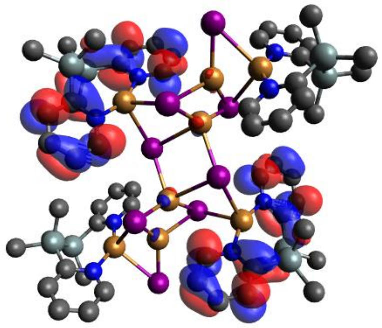	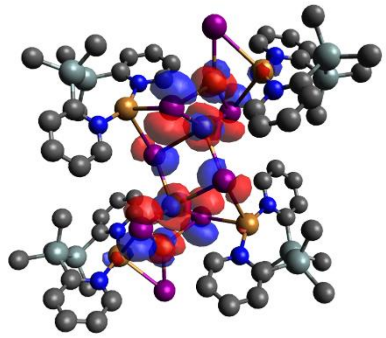
LUMO + 10 (−0.555 eV)	HOMO − 2 (−4.417 eV)
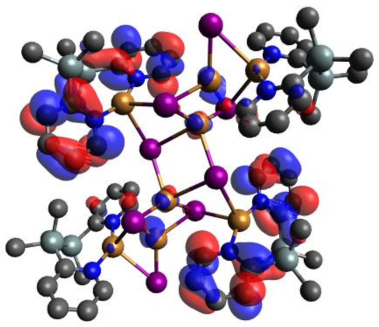	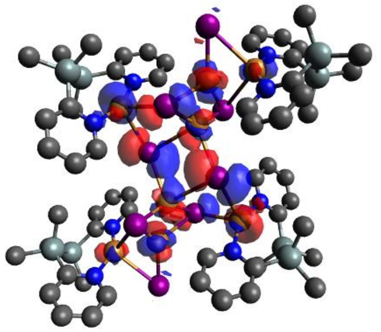
LUMO + 4 (−1.112 eV)	HOMO − 3 (−4.490 eV)
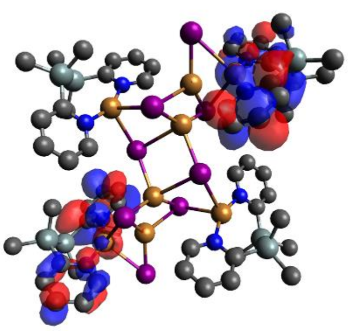	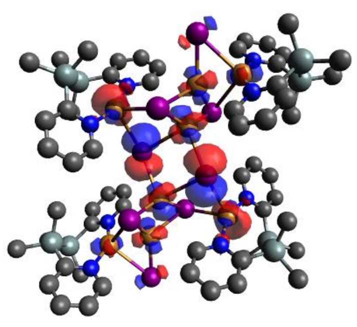
LUMO + 3 (−1.287 eV)	HOMO − 4 (−4.579 eV)

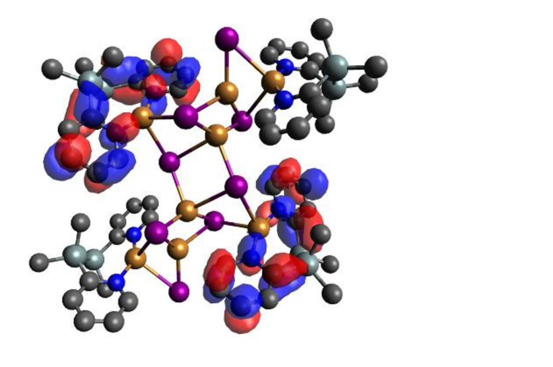

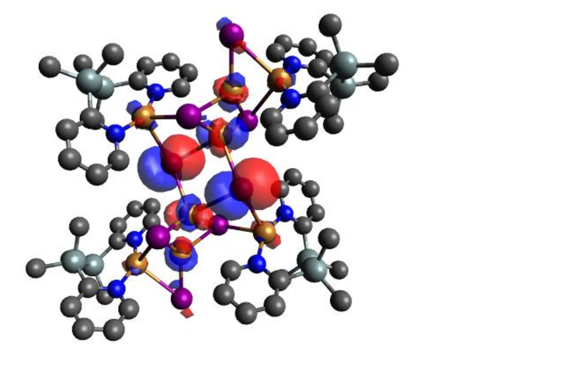

Table S3. Calculated electronic transitions of the octacopper complex **3** obtained by TD-DFT calculation at the B3LYP/LANL2DZ (for iodine) and 6-31G(d) (for others) level.

State[a]	Component (coefficient)	*E*/eV	λ/nm	*f*
T1	H − 2 → L + 14 (0.11412)<br>H − 1 → L (0.10936)<br>H − 1 → L + 4 (0.14154)<br>H − 1 → L + 11 (−0.12680)<br>H − 1 → L + 12 (−0.21800)<br>H − 1 → L + 15 (−0.34601)<br>H → L + 5 (0.13950)<br>H → L + 10 (0.18020)<br>H → L + 13 (0.19284)<br>H → L + 14 (0.32365)	1.9467	636.89	0.0000
T2	H − 2 → L + 15 (−0.11827)<br>H − 1 → L + 5 (0.14670)<br>H − 1 → L + 10 (0.19194)<br>H − 1 → L + 13 (0.20668)<br>H − 1 → L + 14 (0.34295)<br>H → L (0.11156)<br>H → L + 4 (0.13248)<br>H → L + 11 (−0.11885)<br>H → L + 12 (−0.20354)<br>H → L + 15 (−0.32485)	1.9473	636.69	0.0000
T3	H − 2 → L + 1 (0.12617)<br>H − 1 → L (−0.44935)<br>H → L + 1 (0.48544)	2.1043	589.18	0.0000
T4	H − 2→ L (0.12566)	2.1045	589.15	0.0000

	H − 1 → L + 1 (−0.44607)<br>H→ L (0.48741)			
S1	H − 2→ L (0.12566)<br>H − 1 → L + 1 (−0.44607)<br>H→ L (0.48741)	2.1106	587.44	0.0000
S2	H − 2→ L + 1 (0.11885)<br>H − 1 → L (−0.46450)<br>H→ L + 1 (0.50427)	2.1110	587.32	0.0033
T5	H − 6 → L + 3 (−0.15241)<br>H − 5 → L + 2 (0.17695)<br>H − 3→ L + 2 (−0.22317)<br>H − 2 → L + 3 (−0.22355)<br>H → L + 3 (0.53957)	2.2505	550.92	0.0000
S3	H − 5 → L + 3 (0.12117)<br>H − 3 → L + 3 (−0.15512)<br>H − 2 → L + 2 (−0.13315)<br>H − 1 → L + 1 (0.11640)<br>H → L (0.11592)<br>H → L + 2 (0.61493)	2.2954	540.14	0.0000
S4	H − 5 → L + 2 (0.11778)<br>H − 3 → L + 2 (−0.15241)<br>H − 2 → L + 3 (−0.12816)<br>H − 1 → L + 2 (−0.10319)<br>H → L + 3 (0.62698)	2.3023	538.52	0.0124
S5	H − 6 → L (0.10028)<br>H − 3 → L + 1 (0.13729)<br>H − 2 → L (−0.20537)<br>H − 1 → L + 1 (0.41435)<br>H → L (0.44390)<br>H → L + 2 (−0.16667)	2.3348	531.03	0.0000

[a] S and T represents singlet and triplet excited states, respectively.
